# Adding programs and system costs: the need for accurate estimates of the true costs of sexual and reproductive health and rights service provision

**DOI:** 10.1080/26410397.2022.2128748

**Published:** 2022-10-24

**Authors:** Naomi Lince-Deroche, Elizabeth A. Sully, Howard S. Friedman

**Affiliations:** a Independent Consultant, Athens, Greece; b Senior Research Scientist, Guttmacher Institute, New York, NY, USA; c Technical Specialist, Technical Division, United Nations Population Fund (UNFPA), New York, NY, USA

**Keywords:** sexual and reproductive health and rights, health systems, costing, health economics, health policy

## Introduction

Comprehensive sexual and reproductive health and rights (SRHR) programs include an array of services meant to address individuals’ needs throughout their life course. Access to these services is essential for sustainable development. Sustainable Development Goal (SDG) 3/Target 3.7 calls for, “universal access to sexual and reproductive health care services, including for family planning, information and education, and the integration of reproductive health into national strategies and programs” by 2030. Yet maintaining and increasing access have been challenged by the COVID-19 pandemic’s impact on service provision, health care financing and planning.^1^

Efforts to achieve universal access to SRHR are aligned with parallel efforts to achieve another SDG target: universal health coverage (UHC) by 2030. Charting a path toward UHC requires an understanding of current coverage and costs, a strategy that sets targets for 2030, and a process for priority setting and progressive realisation given budget constraints. While establishing expectations for patient-level service delivery and its costs, it is also important to understand how service delivery is dependent on health programs and the health system more broadly.

**Figure 1.**
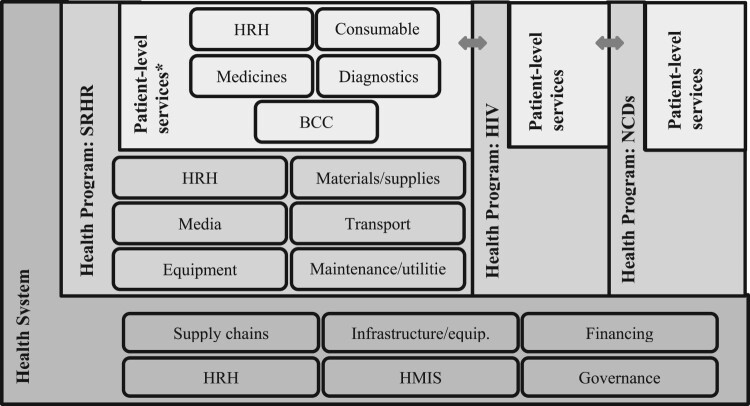
Cost contributors: patient-level services, health programs and health systems Source*:* Adapted from [2,7]. NB: Bidirectional arrows denote service integration. BCC = Behaviour change communication; HRH = Human resources for health; Equip. = equipment; HMIS = Health management information systems. *Patient-level SRHR services would include varying inputs for different services, e.g. family planning, safe abortion, maternal health, etc.

Health systems encompass health condition-specific programs and the cross-cutting systems and support structures required for patient-level service delivery (see Figure 1). Health systems include all the public and private institutions and resources mandated to “improve, maintain, or restore health” within a given geographic and political location.^2^ In contrast, health programs have been defined as the narrower “set of interventions that contribute to the prevention and control of a common health outcome – for example, HIV or non-communicable diseases (NCDs).”^2^ This includes the structures and activities directed towards enhancing program quality, such as training, supervision visits, and monitoring and evaluation.

Country data on programs and system costs as a proportion of overall health care investment needs is difficult to find in the published literature. However, some international costing activities have shown that health programs and system costs may represent the majority of needed incremental investments. A 2017 analysis using the *OneHealth Tool* (OHT) to estimate the costs of achieving the health-related SDGs in low- and middle-income countries (LMICs) found that 75% of needed investments would be for programs and system elements, the largest being the health workforce and infrastructure.^3^ For SRHR, in 2019, programs and system costs were estimated to comprise 62% of the total projected costs of meeting all needs for modern contraception and 65% of total costs for meeting need for pregnancy-related and newborn care in all LMICs.^4^

Despite the importance of programs and system elements, historically, these costs have often been excluded from costing activities and economic evaluations for SRHR, often due to missing information and methodological complexity.^5^ We highlight continued gaps in current practice and outline the potential pitfalls of not including programs and system elements when planning and budgeting for increased access to health care services. We then point out where tools and data exist to improve current practice. Finally, we aim to further efforts targeting universal access to SRHR and for building back better after the COVID-19 pandemic through recommendations for improvements in planning and budgeting for SRHR.

## Program and system gaps in SRHR planning and budgeting

National policies and guidelines for SRHR set a standard for the provision of care and establish pathways to accessing that care. Unfortunately, many of these policies fall short of explicitly acknowledging the necessary programs and system structures and processes required for maintaining successful SRHR service delivery, and likewise the costs of building, scaling or adapting those critical programs and system elements. Within countries, a failure to plan for these costs can mean missed opportunities to improve health care quality, equity, efficiency, accountability and resilience,^6^ whether the exercise is done at the national or sub-national level.

Health policy formulation and costing activities also take place at the global level. These efforts are generally meant to provide normative guidance to countries and to serve as the basis for advocating for greater resource mobilisation. Here too, missing data have hampered efforts to include program and system costs. Of course, the exclusion of these costs may also occur intentionally. Seemingly lower costs may bolster appeals for investment, and when used in cost-effectiveness and cost–benefit computations, lower costs can give the false impression of higher returns on investment than might be realised. Regardless, the result is gaps in guidance and underestimates of true resource needs.

## Tools for improving current practice

Ingredients-based costing in health care is recommended by WHO-CHOICE.^7^ This approach requires identifying all necessary resources and their required quantities (or units), valuing each unit, and then multiplying the quantities by the cost per unit of each resource. Within SRHR, patient-level services include human resources for health, consumables, medicines, diagnostics and behaviour change communication (Figure 1). These staffing and consumable inputs would vary for family planning, safe abortion, maternal health and other SRHR services. However, on top of these patient-level services are the health program costs specific to SRHR, as well as the larger overall health system costs. Program support includes non-client-specific activities and inputs such as health care workers, materials and supplies, equipment, and overhead and administrative costs. At the health system level, one finds the supply chains, health management information systems, governance structures and other entities that form the backbone of all health programs.

Ingredients-based costing can be challenging for programs- and system-level elements of care. The necessary number of inputs per patient is not always clear, and unit costs are often poorly documented. However, there are existing tools to aid programs and system cost calculations. The OneHealth Tool can be used for costing across the health system. The OneHealth Tool was developed to inform sector-wide national strategic health plans and policies by providing information on costs, impact, budgeting and financing gaps with respect to specific coverage scenarios.^8^ The tool includes a module for SRHR and allows for a customisable ingredients approach to individual services, program activities and system elements.^9^ For example, the human resources costing module allows for cadre-specific input of salary, benefits, retention rates, pre-service training capacity, in-service training costs, retention incentives, and recruitment patterns into the public and private sectors.

The OHT’s scope and level of customisability offer an advantage for planning and budgeting. However, true to an ingredients-based approach, the tool requires a significant amount of data. Unfortunately, country-specific cost inputs are frequently lacking. There is a need for improved collection and documentation of costing data in LMICs, especially for health programs and system components of care, and improved sharing or visibility of this data where they do exist. Health cost data repositories can be useful in this regard. For example, the Global Health Cost Consortium maintains a repository of HIV and tuberculosis unit costs (https://ghcosting.org/). Efforts to impute missing data are also helpful. WHO-CHOICE maintains a database of program support costs.^7^ It includes imputed salaries and other input prices, by country. Similar repositories or databases are lacking, especially for SRHR. Here, databases are generally limited to supplies and commodities, such as those provided by UNFPA and UNICEF (https://www.unfpaprocurement.org/products, https://supply.unicef.org/).

The use of mark-up rates is another alternative to add programs and system costs onto a specific direct service or intervention cost. With mark-up rates, the added cost of programs and system elements is assumed to be a proportion of direct costs (*i.e.* direct cost * (1 + *x*% mark-up) = program and system cost). For example, the *Adding It Up* (AIU) analytic model uses ingredients costing for direct service delivery and mark-up rates for programs and system costs. The model aims to estimate the costs and benefits of investing in contraception, pregnancy- and newborn-related care, and sexually transmitted infection services across LMICs.^4^ AIU’s mark-up rates are regionally-specific and based on calculations meant to reflect the *combined* costs of program management, staff supervision, monitoring and evaluation, human resources development, transport and telecommunications, health education and outreach, advocacy, infrastructure and equipment, commodity supply systems, and health information systems.^10^

Mark-up rates are useful for global costing – though less so for individual countries where costs should reflect concrete local circumstances and where costing outputs are used to inform local budgets and resource allocation. That said, even at the global level, for mark-up rates to be useful, they must represent current programs and system costs, including recurring and fixed elements. These rates are dependent on several factors including current and target coverage, capital investment requirements, capacity to pay, local costs of services and other country-specific factors including geography, population size and degree of economic development. Mark-up rates are also dependent on more granular data. Because many components of mark-up rates vary by country and over time, there is a need for efforts to regularly synthesise the granular data into mark-up rates.

## Conclusion and recommendations

Effective health system planning and budgeting, to build back better after the COVID-19 pandemic and to achieve the SDGs, requires an understanding of needs and costs for service delivery and the deeper programs and system-level components that support service delivery. In Box 1, we offer several recommendations for shifting SRHR planning and budgeting specifically. The expectation is that these activities should be undertaken by academic modellers, budget specialists and the range of others involved in these processes. This shift will require support at the country level and in international efforts, including additional resources. It will also require better cost data. There is a need for a global repository of SRHR health care costs, including the ingredients required for direct service provision and the necessary programs and system costs. Finally, these efforts would benefit from the organised support of funders and those involved in leading international costing efforts. The HIV Modeling Consortium (http://hivmodeling.org/), an international consortium of HIV cost modellers supported by donor funding, is an example. They have produced best practice guidelines for costing and work with funders to ensure awareness of best practice.

**Box 1.** Recommendations for improving SRHR planning and budgeting to address the critical role of programs and system costs in ensuring universal access

1. Domestic and international SRHR policy planning and budgeting should explicitly acknowledge the necessary programs and system elements required for successful service delivery, and likewise the costs of building, scaling, or maintaining programs and system elements.2. There is a need for improved collection, documentation, sharing and utilisation of SRHR expenditure and costing data in LMICs especially for health programs and system components of care. This includes a need for standardised approaches, greater methodological transparency and data visibility.3. In the meantime, international repositories of SRHR programs and health system costs should be strengthened and made accessible for imputation purposes.4. Where domestic tools are lacking, the *OneHealth Tool* should be viewed as an off-the-shelf option for estimating health care costs that include programs and system costs to ensure outcomes that reflect full costs.5. Mark-up rates offer an alternative to ingredients-based programs and system costing; however, their use should be limited beyond multi-country or global estimates. Also, they must be kept current and require country-level data for verification.

Improved costing of SRHR services will make clear the resources required to offer rights-based, comprehensive SRHR services. When planning and budgeting for SRHR are informed by estimates of the full costs necessary for service provision, SRHR investments will also contribute to the strengthening of the cross-cutting health programs and system elements necessary for the sustained, resilient provision of all essential health care.
